# Meta-analysis: Early Age at Natural Menopause and Risk for All-Cause and Cardiovascular Mortality

**DOI:** 10.1155/2021/6636856

**Published:** 2021-03-15

**Authors:** Luyao Huan, Xiangling Deng, Mengyang He, Shunhong Chen, Wenquan Niu

**Affiliations:** ^1^Graduate School, Beijing University of Chinese Medicine, Beijing, China; ^2^Department of Emergency, Affiliated Nanping First Hospital, Fujian Medical University, Nanping, Fujian, China; ^3^Institute of Clinical Medical Sciences, China-Japan Friendship Hospital, Beijing, China

## Abstract

**Aims:**

The aim of this meta-analysis was to comprehensively evaluate the association of early age at natural menopause with the risk for all-cause and cardiovascular mortality.

**Methods:**

Literature retrieval was done on August 4, 2020. Article selection and data extraction were completed independently and in duplicate. Early age at natural menopause was grouped into premature menopause (<40 years), early menopause (40-44 years), and relatively early menopause (45-49 years). Effect-size estimates are summarized as hazard ratio (HR) or relative risk (RR) with 95% confidence interval (CI).

**Results:**

Sixteen articles involving 321,233 women were meta-analyzed. Overall analyses revealed a statistically significant association of early age at natural menopause with all-cause mortality risk (HR_adjusted_ = 1.08, 95% CI: 1.03 to 1.14, *P* = 0.002; RR_adjusted_ = 1.05, 95% CI 1.01 to 1.08, *P* = 0.005), but not with cardiovascular mortality risk. In dose-response analyses, the association with all-cause mortality was significant for premature menopause with (HR_adjusted_ = 1.10; 95% CI: 1.01 to 1.21; *P* = 0.034) and without (RR_adjusted_ = 1.34; 95% CI: 1.08 to 1.66; *P* = 0.007) considering follow-up intervals. As for cardiovascular mortality, marginal significance was noted for premature menopause after considering follow-up intervals (HR = 1.09; 95% CI: 1.00-1.19; *P* = 0.045). Subgroup analyses indicated that gender, country, and follow-up periods were possible causes of heterogeneity. There was an overall low probability of publication bias.

**Conclusions:**

Our findings indicate that premature menopause is a promising independent risk factor for both all-cause and cardiovascular mortality.

## 1. Introduction

Menopause is defined as the cessation of spontaneous menses for 12 months, marking the end of a woman's reproductive life [1], and it typically occurs between the ages of 49 and 52 years [2]. An estimated 5% women experience early menopause (menopause onset within 40 to 45 years of age) [3], and 1% women experience premature menopause (menopause onset before 40 years of age) [4, 5]. It is well known that the onset age of menopause is an indicator of reproductive aging, general health, and somatic aging [6]. Evidence from epidemiologic and clinical studies has demonstrated that early age at natural menopause is associated with an enhanced risk for all-cause and cardiovascular mortality [7, 8]. In 2016, Gong and colleagues conducted a meta-analysis of 10 articles, by showing that women who experienced the earliest age natural menopause had a slightly increased all-cause mortality risk [8]. Another review by Muka and colleagues recorded an enhanced risk of cardiovascular mortality and overall mortality in women who had experienced premature or early-onset menopause [9]. Although the association between early age at natural menopause and mortality has been widely evaluated in current medical literature [9–12], there is no definite consensus on this association, possibly due to populations of different races or ethnicities, individually underpowered studies, and lack of adjusting for confounding factors. Given the accumulating data after the year 2016 [8, 9, 13], a more comprehensive evaluation of this association and exploration of possible reasons behind previously inconsistent reports are necessary.

To fill this gap in knowledge and yield more information for future research, we conducted a comprehensive meta-analysis by synthesizing the results of prospective studies that have evaluated the association of early age at natural menopause with all-cause and cardiovascular mortality.

## 2. Materials and Methods

This meta-analysis was complied with the guidelines of the Preferred Reporting Items for Systematic Reviews and Meta-analyses (PRISMA) statement [14], and the PRISMA checklist is summarized in Supplementary Table 1.

In this meta-analysis, as all data were extracted from previous published studies, ethical approval and informed consent are not required.

### 2.1. Search Strategy

We completed literature search as of August 4, 2020, by reviewing PubMed, HuGE Navigator, EMBASE (Excerpt Medica Database), and Web of Science databases. Only published articles written in the English language were considered in the current meta-analysis. The following medical subject heading terms were used: (premenopausal OR early menopause OR perimenopausal OR premature menopause OR menopause OR late menopause OR natural menopause OR postmenopause OR age at menopause OR menopausal age) [Title/Abstract] AND (cardiovascular risk OR coronary heart disease OR cardiovascular disease OR cardiovascular OR all-cause mortality OR cardiovascular mortality) [Title/Abstract]. The reference lists of retrieved articles were also hand searched for potential missing hits.

Two investigators (L.H. and X.D.) independently reviewed all retrieved articles and carefully assessed preliminary eligibility based on the title and abstract, as well as full text when necessary.

### 2.2. Inclusion and Exclusion Criteria

We restricted our analysis to articles that fulfilled the following inclusion criteria: (i) natural menopause; (ii) without estrogen therapy; (iii) multivariate-adjusted hazard ratio (HR) or relative risk (RR) with 95% confidence interval (CI) for quantifying the association of age at natural menopause with cardiovascular or all-cause mortality; (iv) prospective design; (v) all-cause mortality verified by death certificates or medical records; (vi) study subjects free of major underlying diseases.

Articles were excluded if they focused on treatment, survival, or surgical menopause, or lacked control groups, or if they were case report or case series, editorial, narrative review, letter to the editor or correspondence, and non-English articles.

### 2.3. Data Extraction

Two investigators (L.H. and X.D.) independently extracted data from each qualified article, which were typed into a standardized Excel spreadsheet, including the following items: name of first author, publication year, country where study was conducted, sample size, method for mortality ascertainment, baseline age, follow-up period, study design, study type, numbers of deaths, effect-size estimate, and other traditional risk factors, where available, including education, body mass index (BMI), and estrogen therapy. Any disagreement was resolved by a joint reevaluation of original article and, when necessary, was adjudicated by a third author (W. N.).

### 2.4. Quality Appraisal

The quality of the included cohort studies was appraised by means of the NOS (Newcastle-Ottawa Quality Assessment Scale) [15], which is calculated on the basis of three major components: selection of the groups of study (0-4 points), quality of the adjustment for confounding (0-2 points), and ascertainment of the exposure or outcome of interest in the case-control or cohorts, respectively (0-3 points). The maximum score is 9 points, which represents the highest methodological quality.

### 2.5. Statistical Analyses

We employed the STATA software (Stata Corp, College Station, TX, version 14.1 for Windows) for statistical analyses. The effect-size estimates for the association of early age at natural menopause with the risk for cardiovascular or all-cause mortality were summarized as HR or RR with 95% CI under the random-effects models.

The inconsistency index (*I*^2^) was used to assess between-study heterogeneity, and it represents the per cent of observed diversity between studies that is a consequence of heterogeneity other than a chance observation. Significant heterogeneity is recorded if the *I*^2^ is over 50%, and a higher value denotes a higher degree of heterogeneity.

As there are various sources of heterogeneity, a wide panel of subgroups analyses were conducted by region, follow-up period, and age at menopause, respectively. We quantified the probability of publication bias by means of the Begg's funnel plots [16] and Egger regression asymmetry tests [17]. The trim-and-fill method was also used to estimate the number of potentially missing studies in the current meta-analysis. Significant publication bias is recorded if the probability of Egger's tests is below 10%.

## 3. Results

### 3.1. Eligible Studies

After searching four public databases using predefined subject heading terms, we obtained a total of 4,472 articles, and 16 of them involving 321,233 women that assessed the association of early age at natural menopause with all-cause or cardiovascular mortality were eligible for inclusion in the current meta-analysis [12, 18–31].

**Figure 1:**
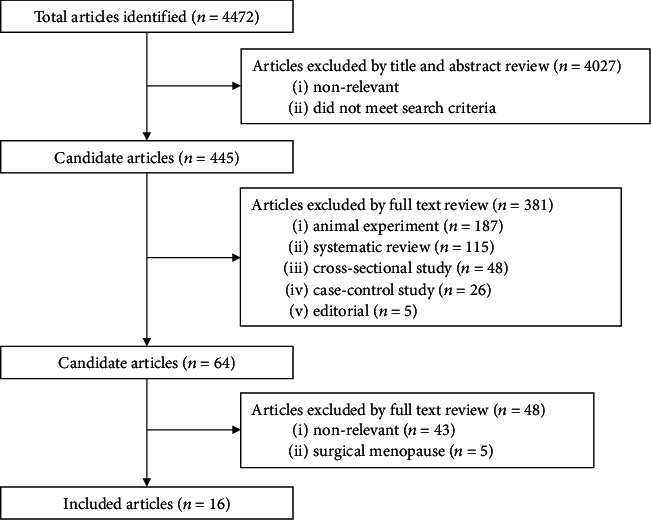
Flow chart of records retrieved, screened, and included in this meta-analysis.

The detailed selection process is presented in Figure 1.

### 3.2. Study Characteristics

Out of 16 eligible articles, only cardiovascular mortality was reported by 2 articles [25, 26], all-cause mortality by 7 articles [19, 23, 24, 27–29, 31], and both by 7 articles [10, 12, 18, 20–22, 30]. Ten studies [10, 18–23, 25, 26, 30] reported HR as effect-size estimates, and RR by 6 studies [12, 24, 27–29, 31], as shown in Table 1. According to statistical methods used, 14 articles [10, 12, 18–23, 25–27, 29–31] used Cox proportional hazards model, 1 article [24] used Poisson regression procedures, and 1 article [28] used logistic regression model (Table 2). The NOS scores ranged from 5 to 8 (Table 3).

According to age at menopause, we divided study subjects into four subgroups: (i) younger than 40 years (premature menopause); (ii) 40-44 years (early menopause); (iii) 45-49 years (relatively early menopause); (iv) 49-52 years (reference category). According to median follow-up periods (in years), we divided data into two subgroups: (i) less than 13.8 years (HR) [19, 22, 23, 25, 30], less than 16.5 years (RR) [12, 24, 29]; (ii) more than or equal to 13.8 years (HR) [10, 18, 20, 21, 26], more than or equal to 16.5 years (RR) [27, 28, 31]. In terms of location, we grouped studies into three groups: America [12, 19–22, 24, 27–29], Europe [26, 31], and Asian [10, 18, 23, 25, 30].

### 3.3. Overall Analyses

After pooling the results of all eligible articles together, we observed a statistically significant association of early age at natural menopause with an increased risk for all-cause mortality (unadjusted HR = 1.12, 95% CI: 1.05 to 1.19, *P* < 0.001; adjusted HR = 1.08, 95% CI: 1.03 to 1.14, *P* = 0.002; unadjusted RR = 1.03, 95% CI: 1.00 to 1.06, *P* = 0.026; adjusted RR = 1.05, 95% CI: 1.01 to 1.08, *P* = 0.005) (Table 4). By contrast, no statistical significance was observed for the association between early age at natural menopause and cardiovascular mortality (unadjusted HR = 1.04, 95% CI: 1.00 to 1.13, *P* = 0.385; adjusted HR = 1.01, 95% CI: 0.95 to 1.09, *P* = 0.682; adjusted RR = 1.08, 95% CI: 0.77 to 1.51, *P* = 0.652) (Table 4). For all-cause mortality, there was no heterogeneity with the *I*^2^ of 45.6% for HR, but there was significant heterogeneity with the *I*^2^ of 60.7% for RR. For cardiovascular mortality, there was no significant evidence of heterogeneity, with the *I*^2^ of 42.1% for HR and 0.0% for RR.

### 3.4. Publication Bias

**Figure 2:**
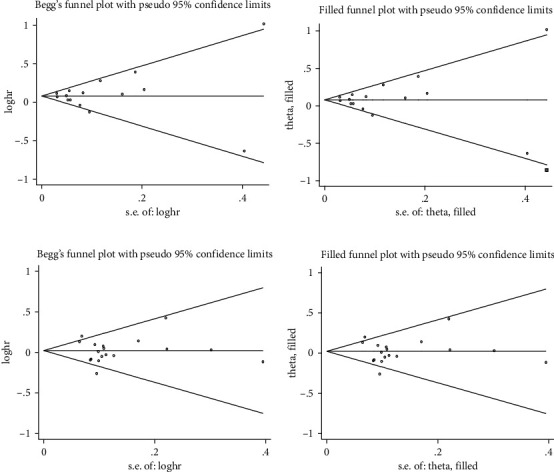
The Begg's and filled funnel plots on the association of early age at natural menopause with all-cause and cardiovascular mortality in studies reporting hazard ratio (HR) as effect-size estimates. (a) Begg's funnel plot and (b) filled funnel plot: all-cause mortality (HR). (c) Begg's funnel plot and (d) filled funnel plot: cardiovascular mortality (HR).

The possibility of publication bias was illustrated using the Begg's funnel plots for the association of early age at natural menopause with all-cause (Figure 2) and cardiovascular (Supplementary Figure 1) mortality, respectively, and they seemed symmetric. As further revealed by the Egger's tests, in studies reporting HR, there was no evidence of publication bias for all-cause mortality (*P* = 0.746) and cardiovascular mortality (*P* = 0.782). By contrast, in studies reporting RR, there was strong evidence of publication bias for all-cause mortality (*P* = 0.010), yet no publication bias for cardiovascular mortality (*P* = 0.456).

As reflected by the filled funnel plots, one and six additional studies were separately required for the relationship between all-cause mortality and early age at natural menopause in studies reporting HR and RR. For cardiovascular mortality, no study was missing for studies reporting HR and RR.

After adjusting for potential missing studies, effect-size estimates were still statistically significant for all-cause mortality (HR = 1.08, 95% CI: 1.03 to 1.14, *P* = 0.002). On the contrary, even if the funnel plots were further filled to make the cardiovascular mortality plot symmetric, after adjustment for potential missing studies, effect-size estimates were not statistically significant for cardiovascular mortality (HR = 1.01, 95% CI: 0.95 to 1.09, *P* = 0.682; RR = 1.08, 95% CI: 0.77 to 1.51, *P* = 0.652).

### 3.5. Subgroup Analyses

We subsequently conducted a large panel of subgroup analyses, because the *I*^2^ suggested the possible existence of between-study heterogeneity (Table 4).

By geographic location, in studies reporting HR, the association between early age at natural menopause and all-cause mortality was statistically significant in Asian countries (HR = 1.11, 95% CI: 1.03 to 1.18, *P* = 0.004), but not in American countries (HR = 1.05, 95% CI: 0.96 to 1.15, *P* = 0.268). In studies reporting RR, the association between early age at natural menopause and all-cause mortality was statistically significant in both American countries (RR = 1.08, 95% CI: 1.02 to 1.15, *P* = 0.010) and European countries (RR = 1.03, 95% CI: 1.01 to 1.08, *P* = 0.010) (Two-sample *Z* test *P* = 0.088). However, the association between early age at natural menopause and cardiovascular mortality was not statistically significant in American (HR = 1.06, 95% CI: 0.97 to 1.16, *P* = 0.235; RR = 1.08, 95% CI: 0.77 to 1.51, *P* = 0.652), Europe (HR = 0.85, 95% CI: 0.71 to 1.01, *P* = 0.060), and Asian (HR = 1.05, 95% CI: 0.97 to 1.14, *P* = 0.228) countries.

In studies reporting HR, by the median value (13.8 years) of follow-up intervals, the association of early age at natural menopause with all-cause mortality was significant in both short (<13.8 years) (HR = 1.09, 95% CI: 1.00 to 1.18, *P* = 0.047) and long (≥13.8 years) (HR = 1.08, 95% CI: 1.01 to 1.16, *P* = 0.036) follow-ups. By contrary, in studies reporting RR, by the median value (16.5 years) of follow-up intervals, the association of early age at natural menopause with all-cause mortality was significant in short (<16.5 years) (RR = 1.21, 95% CI: 1.03 to 1.41, *P* = 0.020), but not in long (≥16.5years) (RR = 1.08, 95% CI: 0.77 to 1.51, *P* = 0.652) follow-ups. The association of early age at natural menopause with cardiovascular mortality was not significant in long (≥13.8 years) (HR = 1.01, 95% CI: 0.90 to 1.14, *P* = 0.811) and short (<13.8 years) (HR = 1.02, 95% CI: 0.95 to 1.10, *P* = 0.560) follow-ups in studies reporting HR. Meanwhile, in studies reporting RR, the association of early age at natural menopause with cardiovascular mortality was not significant.

### 3.6. Dose-Response Analyses

In the dose-response analysis of all-cause mortality in studies reporting HR, the association of premature menopause with all-cause mortality was significant (HR = 1.10, 95% CI: 1.01 to 1.21, *P* = 0.034), but not for early (HR = 1.12, 95% CI: 0.96 to 1.31, *P* = 0.145) and relatively early menopause (HR = 1.05, 95% CI: 1.00 to 1.10, *P* = 0.051). In studies reporting RR, women with premature menopause (RR = 1.34, 95% CI: 1.08 to 1.66, *P* = 0.007) had a higher risk than women with relatively early menopause (RR = 1.02, 95% CI: 1.01 to 1.04, *P* = 0.004) (Two-sample *Z* test *P* = 0.010) for all-cause mortality.

In the dose-response analysis of cardiovascular mortality in studies reporting HR, the association was only significant for premature menopause (HR = 1.09, 95% CI: 1.00 to 1.19, *P* = 0.045). However, in studies reporting RR, no statistical significance was observed across age groups in cardiovascular mortality (<40 years: RR = 1.26, 95% CI: 0.56 to 2.85, *P* = 0.579; 40-44 years: RR = 1.04, 95% CI: 0.54 to 2.00, *P* = 0.906; 45-49 years: RR = 1.05, 95% CI: 0.67 to 1.64, *P* = 0.830).

## 4. Discussion

Via a comprehensive meta-analysis of 16 articles and 321,233 women, our findings indicate that premature menopause is a promising independent risk factor for both all-cause and cardiovascular mortality. Moreover, gender, country, and follow-up periods were identified as possible causes of between-study heterogeneity. To the best of our knowledge, this is the most comprehensive report that has meta-analyzed the prediction of early age at natural menopause for all-cause and cardiovascular mortality risk.

In 2016, Gong and colleagues in a meta-analysis of 10 articles investigated the association of early age at natural menopause with all-cause and cardiovascular mortality risk, and they found that the earliest age at natural menopause (<40 years) was associated with a slightly increase in all-cause mortality, but not with cardiovascular and stroke-related mortality [8]. In addition, they also found that women with natural menopause before 40 years of age had an 18% greater risk of all-cause mortality, and this relationship was not statistically significant in women with natural menopause before age 46.7 years [8]. The findings of the current meta-analysis on 16 articles reinforced that of the meta-analysis by Gong and colleagues [8], by reinforcing that early age at natural menopause was associated with a slightly increased risk of all-cause mortality. However, by further subdividing age of early menopause into three groups and different reports of study results into HRs and RRs, our findings indicated that premature menopause was more strongly associated with all-cause mortality than early natural menopause and relatively early natural menopause. Extending the findings of the meta-analysis by Gong and colleagues [8], as expected, we reported that early age at natural menopause was not significantly associated with cardiovascular mortality. More importantly, using the relatively large number of eligible articles, we further explored possible causes of between-study heterogeneity by conducting a wide panel of subgroup analyses.

Our finding on the association of natural menopause age before 40 years with increased all-cause mortality risk supports the hypothesis that premature natural menopause may serve as a marker of accelerated reproductive and somatic aging, as well as causing premature death [12]. Meantime, the timing of menopause reflects a complex interplay of genetic, epigenetic, socioeconomic, and lifestyle factors [32]. Several mechanisms have been proposed to interpret the association between earlier menopause and an increased risk of all-cause mortality, and the most promising reason may be lack of estrogen. Estrogens are potent vasoactive hormones that promote vascular remodeling and elasticity, and they can regulate reactive dilation and local inflammatory activity [33], as well as endothelial vasodilator dysfunction after estrogen deficiency [34, 35]. Estrogens also play a key role in the regulation of calcium homeostasis, and thus fine-tuning the normal process of cardiomyocyte contraction and relaxation [36]. Of course, it has also been suggested that this increased risk is associated with an increase in luteinizing hormone [37] or modulation of T cells [38].

Epidemiological studies have shown that premature menopause before 40 years of age affects about 1% of women [4], and as demonstrated in the current meta-analysis, the risk for all-cause mortality was greatest for women with premature menopause. The possible reasons lie in that earlier loss of the ovarian function can lead to long-term activation of the renin-angiotensin-aldosterone system, endothelial dysfunction, inflammation and immune dysfunction, and further acceleration of the occurrence or progression of chronic diseases, ending with the predisposition to all-cause mortality [9, 39]. Furthermore, menopause marks the beginning of a biological mechanism caused by hormonal changes leading to tissue damage and organ dysfunction [40], and premature menopause is the earlier onset of this biological mechanism.

There are several possible limitations for the current meta-analysis. Firstly, because only published articles were retrieved and the “grey” literature (articles in languages other than the English) was not incorporated, publication bias is possible. Additionally, as the number of studies reporting RR as effect-size estimates is less than 10 in this meta-analysis, the power to detect statistical significance is low [41]. Secondly, age at menopause was self-reported, and misclassification of the groups by age at menopause cannot be excluded. Thirdly, women in these studies were limited only to natural menopause, and we did not address the effect of surgical menopause or medical interventions that induced menopause-relevant mortality.

## 5. Conclusions

Our findings indicated that premature menopause is a promising independent risk factor for both all-cause and cardiovascular mortality. To date, the most effective treatment is still hormone replacement therapy, yet this method may increase the incidence of more serious concerns such as venous thromboembolism and cancer [42]. Therefore, investigations on the mechanisms and therapies between early age at menopause and all-cause mortality are also warranted.

## Figures and Tables

**Table 1:** The baseline characteristics of all involved studies in this meta-analysis.

Year	First author	Country	Sample size	Age (years)	Follow-up (years)	Ascertainment of mortality	Total deaths	CVD deaths	Exposure (years)	Ref (years)	Effect estimate	Adjustment	HR (95% CI) (all-cause)	HR (95% CI) (CVD)
2002	Kleijn	Netherlands	9450	35-66	20.5	Death certificates	2439	1063	>44 and ≤48	≤44	HR	No	—	0.78 (0.65-0.94)
2002	Kleijn	Netherlands	9450	35-66	20.5	Death certificates	2439	1063	>44 and ≤48	≤44	HR	Yes	—	0.77 (0.64-0.93)
2002	Kleijn	Netherlands	9450	35-66	20.5	Death certificates	2439	1063	>48 and ≤51	≤44	HR	No	—	0.92 (0.78-1.09)
2002	Kleijn	Netherlands	9450	35-66	20.5	Death certificates	2439	1063	>48 and ≤51	≤44	HR	Yes	—	0.92 (0.78-1.09)
2006	Amagai	Japan	4683	36-89	9.2	Death certificates	215	—	<40	45-49	HR	No	2.75 (1.19-6.36)	—
2006	Amagai	Japan	4683	36-89	9.2	Death certificates	215	—	<40	45-49	HR	Yes	2.77 (1.16-6.58)	—
2006	Amagai	Japan	4683	36-89	9.2	Death certificates	215	—	40-44	45-49	HR	No	0.67 (0.34-1.30)	—
2006	Amagai	Japan	4683	36-89	9.2	Death certificates	215	—	40-44	45-49	HR	Yes	0.53 (0.24-1.17)	—
2006	Cui	Japan	37965	40-79	10	International classification of disease	—	1010	≤44	≥51	HR	No	—	1.07 (0.88-1.32)
2006	Cui	Japan	37965	40-79	10	International classification of disease	—	1010	≤44	≥51	HR	Yes	—	1.08 (0.88-1.34)
2006	Cui	Japan	37965	40-79	10	International classification of disease	—	1010	45-46	≥51	HR	No	—	0.96 (0.77-1.19)
2006	Cui	Japan	37965	40-79	10	International classification of disease	—	1010	45-46	≥51	HR	Yes	—	0.97 (0.78-1.21)
2006	Cui	Japan	37965	40-79	10	International classification of disease	—	1010	47-48	≥51	HR	No	—	1.12 (0.93-1.34)
2006	Cui	Japan	37965	40-79	10	International classification of disease	—	1010	47-48	≥51	HR	Yes	—	1.10 (0.92-1.32)
2006	Cui	Japan	37965	40-79	10	International classification of disease	—	1010	49-50	≥51	HR	No	—	0.92 (0.78-1.09)
2006	Cui	Japan	37965	40-79	10	International classification of disease	—	1010	49-50	≥51	HR	Yes	—	0.91 (0.77-1.97)
2007	Hong	Korea	2658	≥55	15.8	Statistics on the causes of death of Korea	1193	297	<40	45-49	HR	No	1.33 (1.07-1.65)	—
2007	Hong	Korea	2658	≥55	15.8	Statistics on the causes of death of Korea	1193	297	<40	45-49	HR	Yes	1.32 (1.05-1.66)	—
2007	Hong	Korea	2658	≥55	15.8	Statistics on the causes of death of Korea	1193	297	<40	45-49	HR	No	—	1.58 (1.04-2.41)
2007	Hong	Korea	2658	≥55	15.8	Statistics on the causes of death of Korea	1193	297	<40	45-49	HR	Yes	—	1.53 (1.01-2.39)
2007	Hong	Korea	2658	≥55	15.8	Statistics on the causes of death of Korea	1193	297	40-44	45-49	HR	No	1.13 (0.97-1.32)	—
2007	Hong	Korea	2658	≥55	15.8	Statistics on the causes of death of Korea	1193	297	40-44	45-49	HR	Yes	1.13 (0.97-1.34)	—
2007	Hong	Korea	2658	≥55	15.8	Statistics on the causes of death of Korea	1193	297	40-44	45-49	HR	No	—	1.17 (0.84-1.62)
2007	Hong	Korea	2658	≥55	15.8	Statistics on the causes of death of Korea	1193	297	40-44	45-49	HR	Yes	—	1.15 (0.82-1.60)
2012	Tom	USA	1684	≥65	24	National death index	1477	1231	<45	50-54	HR	No	0.89 (0.74-1.07)	—
2012	Tom	USA	1684	≥65	24	National death index	1477	1231	<45	50-54	HR	Yes	0.88 (0.73-1.06)	—
2012	Tom	USA	1684	≥65	24	National death index	1477	1231	<45	50-54	HR	No	—	0.97 (0.76-1.24)
2012	Tom	USA	1684	≥65	24	National death index	1477	1231	<45	50-54	HR	Yes	—	0.96 (0.75-1.23)
2012	Tom	USA	1684	≥65	24	National death index	1477	1231	45-49	50-54	HR	No	0.95 (0.82-1.10)	—
2012	Tom	USA	1684	≥65	24	National death index	1477	1231	45-49	50-54	HR	Yes	0.96 (0.83-1.12)	—
2012	Tom	USA	1684	≥65	24	National death index	1477	1231	45-49	50-54	HR	No	—	0.93 (0.76-1.24)
2012	Tom	USA	1684	≥65	24	National death index	1477	1231	45-49	50-54	HR	Yes	—	0.95 (0.77-1.16)
2014	Wu	China	31955	40-70	11.2	Medical records	3158	1001	<46.64	48.80-50.15	HR	No	1.17 (1.05-1.31)	—
2014	Wu	China	31955	40-70	11.2	Medical records	3158	1001	<46.64	48.80-50.15	HR	Yes	1.16 (1.04-1.29)	—
2014	Wu	China	31955	40-70	11.2	Medical records	3158	1001	<46.64	48.80-50.15	HR	No	—	1.03 (0.85-1.24)
2014	Wu	China	31955	40-70	11.2	Medical records	3158	1001	<46.64	48.80-50.15	HR	Yes	—	1.01 (0.83-1.22)
2014	Wu	China	31955	40-70	11.2	Medical records	3158	1001	46.64-48.79	48.80-50.15	HR	No	1.04 (0.93-1.17)	—
2014	Wu	China	31955	40-70	11.2	Medical records	3158	1001	46.64-48.79	48.80-50.15	HR	Yes	1.03 (0.92-1.15)	—
2014	Wu	China	31955	40-70	11.2	Medical records	3158	1001	46.64-48.79	48.80-50.15	HR	No	—	0.92 (0.76-1.11)
2014	Wu	China	31955	40-70	11.2	Medical records	3158	1001	46.64-48.79	48.80-50.15	HR	Yes	—	0.90 (0.74-1.09)
2018	Lay	Brazil	868	≥70	16	Death certificates	444	199	≤40	50-54	HR	No	1.27 (0.88-1.83)	—
2018	Lay	Brazil	868	≥70	16	Death certificates	444	199	≤40	50-54	HR	Yes	1.18 (0.79-1.76)	—
2018	Lay	Brazil	868	≥70	16	Death certificates	444	199	≤40	50-54	HR	No	—	1.17 (0.65-2.09)
2018	Lay	Brazil	868	≥70	16	Death certificates	444	199	≤40	50-54	HR	Yes	—	1.03 (0.57-1.86)
2018	Lay	Brazil	868	≥70	16	Death certificates	444	199	41-44	50-54	HR	No	1.38 (0.94-2.03)	—
2018	Lay	Brazil	868	≥70	16	Death certificates	444	199	41-44	50-54	HR	Yes	1.48 (1.03-2.14)	—
2018	Lay	Brazil	868	≥70	16	Death certificates	444	199	41-44	50-54	HR	No	—	0.83 (0.38-1.78)
2018	Lay	Brazil	868	≥70	16	Death certificates	444	199	41-44	50-54	HR	Yes	—	0.89 (0.41-1.93)
2018	Lay	Brazil	868	≥70	16	Death certificates	444	199	45-49	50-54	HR	No	1.17 (0.87-1.58)	—
2018	Lay	Brazil	868	≥70	16	Death certificates	444	199	45-49	50-54	HR	Yes	1.11 (0.81-1.52)	—
2018	Lay	Brazil	868	≥70	16	Death certificates	444	199	45-49	50-54	HR	No	—	1.09 (0.72-1.66)
2018	Lay	Brazil	868	≥70	16	Death certificates	444	199	45-49	50-54	HR	Yes	—	1.04 (0.67-1.60)
2019	Malek	USA	11287	≥45	7.1	Social security death index and National Death Index	1524	—	<45	≥45	HR	No	1.17 (1.06-1.30)	—
2019	Malek	USA	11287	≥45	7.1	Social security death index and National Death Index	1524	—	<45	≥45	HR	Yes	1.03 (0.93-1.14)	—
2019	Zhang	USA	75359	50-78	13	Death certificates	7826	1584	≤44	45-54	HR	No	1.24 (1.18-1.30)	—
2019	Zhang	USA	75359	50-78	13	Death certificates	7826	1584	≤44	45-54	HR	Yes	1.12 (1.05-1.18)	—
2019	Zhang	USA	75359	50-78	13	Death certificates	7826	1584	≤44	45-54	HR	No	—	1.32 (1.18-1.47)
2019	Zhang	USA	75359	50-78	13	Death certificates	7826	1584	≤44	45-54	HR	Yes	—	1.14 (1.01-1.30)
2020	Shen	Taiwan	36931	61(mean)	14.6	National Death Index	5316	1141	<40-44	50-54	HR	No	1.10 (1.00-1.21)	—
2020	Shen	Taiwan	36931	61	14.6	National death index	5316	1141	<40-44	50-54	HR	Yes	1.09 (0.99-1.20)	—
2020	Shen	Taiwan	36931	61	14.6	National death index	5316	1141	<40-44	50-54	HR	No	—	1.06 (0.85-1.31)
2020	Shen	Taiwan	36931	61	14.6	National death index	5316	1141	<40-44	50-54	HR	Yes	—	1.05 (0.85-1.30)
2020	Shen	Taiwan	36931	61	14.6	National death index	5316	1141	45-49	50-54	HR	No	1.08 (1.01-1.15)	—
2020	Shen	Taiwan	36931	61	14.6	National death index	5316	1141	45-49	50-54	HR	Yes	1.07 (1.01-1.14)	—
2020	Shen	Taiwan	36931	61	14.6	National death index	5316	1141	45-49	50-54	HR	No	—	1.21 (1.06-1.39)
2020	Shen	Taiwan	36931	61	14.6	National death index	5316	1141	45-49	50-54	HR	Yes	—	1.22 (1.07-1.40)
1998	Cooper	USA	3191	50-86	4	Death certificates	345	—	<40	45-49	RR	Yes	1.56 (1.07-2.27)	—
1998	Cooper	USA	3191	50-86	4	Death certificates	345	—	40-44	45-49	RR	Yes	1.10 (0.80-1.51)	—
1999	Jacobsen	USA	5279	≥25	13	Death certificates	1831	—	35-40	49-51	RR	Yes	1.30 (1.10-1.50)	—
1999	Jacobsen	USA	5279	≥25	13	Death certificates	1831	—	41-44	49-51	RR	Yes	0.90 (0.80-1.10)	—
1999	Jacobsen	USA	5279	≥25	13	Death certificates	1831	—	45-48	49-51	RR	Yes	0.99 (0.90-1.10)	—
2000	Cooper	USA	826	63-81	55	Death certificates	108	—	28-45	≥51	RR	Yes	1.39 (0.63-3.04)	—
2000	Cooper	USA	826	63-81	55	Death certificates	108	—	46-50	≥51	RR	Yes	1.38 (0.86-2.22)	—
2003	Jacobsen	Norway	19731	32-74	37	Official personal registration	18533	—	≤40	50-52	RR	Yes	1.06 (0.99-1.14)	—
2003	Jacobsen	Norway	19731	32-74	37	Official personal registration	18533	—	41-43	50-52	RR	Yes	1.02 (0.96-1.09)	—
2003	Jacobsen	Norway	19731	32-74	37	Official personal registration	18533	—	44-46	50-52	RR	Yes	1.05 (1.01-1.09)	—
2003	Jacobsen	Norway	19731	32-74	37	Official personal registration	18533	—	47-49	50-52	RR	Yes	1.01 (0.98-1.05)	—
2005	Mondul	USA	68154	≥30	20	Death certificates	23067	—	40-44	50-54	RR	No	1.05 (1.01-1.09)	—
2005	Mondul	USA	68154	≥30	20	Death certificates	23067	—	40-44	50-54	RR	Yes	1.04 (1.01-1.08)	—
2005	Mondul	USA	68154	≥30	20	Death certificates	23067	—	45-49	50-54	RR	No	1.02 (0.99-1.05)	—
2005	Mondul	USA	68154	≥30	20	Death certificates	23067	—	45-49	50-54	RR	Yes	1.02 (1.01-1.05)	—
2013	Li	USA	11212	21-69	13	National death index and otification from next of kin and postal	692	199	<40	50-54	RR	Yes	1.97 (1.30-2.99)	—
2013	Li	USA	11212	21-69	13	National death index and notification from next of kin and postal	692	199	<40	50-54	RR	Yes	—	1.26 (0.56-2.86)
2013	Li	USA	11212	21-69	13	National death index and notification from next of kin and postal	692	199	40-44	50-54	RR	Yes	1.50 (1.08-2.06)	—
2013	Li	USA	11212	21-69	13	National death index and notification from next of kin and postal	692	199	40-44	50-54	RR	Yes	—	1.04 (0.54-1.99)
2013	Li	USA	11212	21-69	13	National death index and notification from next of kin and postal	692	199	45-49	50-54	RR	Yes	1.13 (0.88-1.44)	—
2013	Li	USA	11212	21-69	13	National death index and notification from next of kin and postal	692	199	45-49	50-54	RR	Yes	—	1.05 (0.67-1.63)

Ref: reference; RR: relative risk; HR: hazard ratio; CVD: cardiovascular disease.

**Table 2:** The statistical methods of all involved studies in this meta-analysis.

Year	First author	Country	Sample size	Study type	Statistical method	Adjustment for confounders	Effect estimate
2002	Kleijn	Netherlands	9450	Cohort study	Cox proportional hazards model	Age, hormone replacement therapy use, hypertension, BMI, and social economic class.	HR
2006	Amagai	Japan	4683	Cohort study	Cox proportional hazard model	Age, SBP, TC, HDL-C, history of DB, BMI, smoking, alcohol, marital status, study area, and type of menopause.	HR
2006	Cui	Japan	37965	Cohort study	Cox proportional hazard model	Age, smoking, alcohol, marital status, type of menopause, education, hypertension, and diabetes.	HR
2007	Hong	Korea	2658	Cohort study	Cox proportional hazards model	Age, alcohol consumption, education, age at first birth, self-cognitive health level, chronic disease, marital partner, parity, age at menarche, oral contraceptive use, and hypertension.	HR
2012	Tom	USA	1684	Cohort study	Cox proportional hazards model	Age, education, pregnancy number, age at menarche, smoking, height, weight, and use of ET.	HR
2014	Wu	China	31955	Cohort study	Cox proportional hazard model	Age at study enrollment, BMI, WHR, education, occupation, income, regular exercise, current smoking or alcohol, marital status, age at menarche, and number of live births.	HR
2018	Lay	Brazil	868	Cohort study	Cox regression model	Social, year of birth, education, marital status, race, reproductive, parity, smoking, number of chronic diseases, and ET.	HR
2019	Malek	USA	11287	Cohort study	Cox proportional hazards model	Age, race, education, medical conditions, behavioral characteristics, and type of menopause.	HR
2019	Zhang	USA	75359	Cohort study	Cox proportional hazards model	Baseline age, race, BMI, smoking, alcohol, marital status, education level, physical exercise, hormone replacement therapy use, CVD history, number of live births, age at first birth, and type of menopause.	HR
2020	Shen	Taiwan	36931	Cohort study	Cox proportional hazards model	Birth cohort, education, smoking status, body mass index, hypertension, diabetes, and high blood cholesterol.	HR
1998	Cooper	USA	3191	Cohort study	Poisson regression procedures	Age, education, race, smoking, use of estrogen therapy, and years of follow-up.	RR
1999	Jacobsen	USA	5279	Cohort study	Proportional hazards model	DB, hypertension, parity, age at first birth, leisure PA, education, BMI, current use of estrogen, ever-smoking, vegetarian status, and dietary pattern.	RR
2000	Cooper	USA	826	Cohort study	Logistic regression model	Age, smoking, use of estrogen replacement therapy, and parity.	RR
2003	Jacobsen	Norway	19731	Cohort study	Cox proportional hazards model	Attained age, county, occupation, and birth cohort.	RR
2005	Mondul	USA	68154	Cohort study	Cox proportional hazards model	Age, race, marital status, BMI, age at menarche, parity, education, alcohol, oral contraceptive use, and exercise.	RR
2013	Li	USA	11212	Cohort study	Cox proportional hazard model	Age and time period, education, marital status, BMI, smoking, alcohol, PE, dietary pattern, menarche age, parity or first birth, reproductive factors, contraceptive use, lactation duration, and unilateral oophorectomy.	RR

RR: relative risk; HR: hazard ratio; BMI: body mass index; SBP: systolic blood pressure; CVD: cardiovascular disease; HDL-C: high-density lipoprotein cholesterol; PE: physical education; TC: total cholesterol; DB: diabetes mellitus; WHR: waist-to hip ratio; PA: physical activity.

**Table 3:** The Newcastle-Ottawa Scale (NOS) for assessing the quality of cohort studies.

First author	Year	Selection				Comparability		Outcome			
		Representative of the exposed cohort	Selection of the nonexposed cohort	Ascertainment of exposed	Demonstration that outcome of interest was no present at start of study	Control for important cohort	Additional factors	Assessment of outcome	Follow-up	Adequacy of follow-up	Score
Cooper	1998	1	1	0	1	1	1	1	0	0	6
Jacobsen	1999	0	0	1	1	1	1	1	0	0	5
Cooper	2000	0	0	1	1	1	1	1	1	0	6
Kleijn	2002	1	1	0	1	1	1	1	1	1	8
Jacobsen	2003	1	1	1	1	1	1	1	1	0	8
Mondul	2005	1	1	1	1	1	1	1	1	0	8
Amagai	2006	1	1	0	1	1	1	1	0	0	6
Cui	2006	1	1	0	1	1	1	1	0	0	6
Hong	2007	1	1	1	1	1	1	0	1	0	7
Tom	2012	1	1	0	1	1	1	1	1	1	8
Li	2013	0	0	1	1	1	1	1	1	0	6
Wu	2014	1	1	1	1	1	1	1	0	0	7
Lay	2018	1	1	1	1	1	1	1	1	0	8
Malek	2019	1	1	1	1	1	1	0	0	0	6
Zhang	2019	1	1	1	1	1	1	1	1	0	8
Shen	2020	1	1	0	1	1	1	0	1	0	6

**Table 4:** Overall and subgroup analyses of early age at natural menopause with all-cause and cardiovascular mortality.

Group	Studies (n)	All-cause mortality		Cardiovascular mortality	
Effect-size estimate		HR (95% CI); *P*	*I* ^2^	HR (95% CI); *P*	*I* ^2^
Overall analyses					
Mortality (unadjusted)	15/18	1.12 (1.05-1.19); <0.001	65.3%	1.04 (1.00-1.13); 0.385	61.7%
Mortality (adjusted)	15/18	1.08 (1.03-1.14); 0.002	45.6%	1.01 (0.95-1.09); 0.682	42.1%
Subgroup analyses					
By country					
America	7/6	1.05 (0.96-1.15); 0.268	51.5%	1.06 (0.96-1.16); 0.235	0.0%
Europe	NA/2	NA	NA	0.85 (0.71-1.01); 0.060	48.3%
Asia	8/10	1.11 (1.03-1.18); 0.004	46.7%	1.05 (0.97-1.14); 0.228	37.1%
By follow-up					
<13.8 years	6/7	1.09 (1.00-1.18); 0.047	57.7%	1.02 (0.95-1.10); 0.560	20.6%
≥13.8 years	9/11	1.08 (1.01-1.16); 0.036	40.0%	1.01 (0.90-1.14); 0.811	54.0%
Dose-response analysis					
<40 years	7/6	1.10 (1.01-1.21); 0.034	60.7%	1.09 (1.00-1.19); 0.045	0.0%
40-44 years	4/3	1.12 (0.96-1.31); 0.145	49.2%	1.07 (0.90-1.27); 0.464	0.0%
45-49 years	4/9	1.05 (1.00-1.10); 0.051	0.0%	0.97 (0.88-1.07); 0.539	60.8%
Effect-size estimate		RR (95% CI); *P*	*I* ^2^	RR (95% CI); *P*	*I* ^2^
Overall analyses					
Mortality (unadjusted)	2/NA	1.03 (1.00-1.06); 0.026	28.2%	NA	NA
Mortality (adjusted)	16/3	1.05 (1.01-1.08); 0.005	60.7%	1.08 (0.77-1.51); 0.652	0.0%
Subgroup analyses					
By country					
America	12/3	1.08 (1.02-1.15); 0.010	68.8%	1.08 (0.77-1.51); 0.652	0.0%
Europe	4/NA	1.03 (1.01-1.08); 0.010	0.0%	NA	NA
Asia	NA/NA	NA	NA	NA	NA
By follow-up					
<16.5 years	8/8	1.21 (1.03-1.41); 0.020	76.0%	1.08 (0.77-1.51); 0.652	0.0%
≥16.5 years	3/NA	1.08 (0.77-1.51); 0.652	0.0%	NA	NA
Dose-response analysis					
<40 years	5/1	1.34 (1.08-1.66); 0.007	75.3%	1.26 (0.56-2.85); 0.579	NA
40-44 years	5/1	1.03 (0.96-1.10); 0.377	52.7%	1.04 (0.54-2.00); 0.906	NA
45-49 years	6/1	1.02 (1.01-1.04); 0.004	0.0%	1.05 (0.67-1.64); 0.830	NA

HR: hazard ratio; RR: relative risk; 95% CI: 95% confidence interval; NA: not available.

## Abbreviations

CVD: Cardiovascular disease
EMBASE: Excerpt Medica Database
HR: Hazard ratio
RR: Risk ratio
CI: Confidence interval
*I^2^*: Inconsistency index
PRISMA: Preferred Reporting Items for Systematic Reviews and Meta-analyses.

## Supplementary Materials

Supplementary Materials Supplementary Table 1: the PRISMA checklist for all included studies in this meta-analysis. Supplementary Figure 1: Begg's and filled funnel plots on the association of early age at natural menopause with all-cause and cardiovascular mortality in studies reporting relative risk (RR) as effect-size estimates.
